# Editorial for acute mesenteric ischemia: Novel diagnostic modalities and treatment strategies to improve patient outcome

**DOI:** 10.3389/fsurg.2023.1205970

**Published:** 2023-04-28

**Authors:** Matthias Mehdorn

**Affiliations:** Department of Visceral, Transplantation, Thoracic and Vascular Surgery, University Hospital Leipzig, Leipzig, Saxony, Germany

**Keywords:** mesenteric ischemia, bowel obstruction, SMA dissection, HMGB1, myoglobin

**Editorial on the Research Topic** Acute mesenteric ischemia: Novel diagnostic modalities and treatment strategies to improve patient outcome

In many fields of modern medical diagnostics and treatment, drastic improvements in patient outcome can be seen, i.e., in oncology with immune therapy. Unfortunately, acute mesenteric ischemia remains a life-threatening condition due to the uncharacteristic signs and symptoms with subsequently delayed diagnostics. Thus, the diagnosis is often found too late and nothing but extensive bowel resection remains as only possible therapeutic option. The search for serum parameters in acute ischemia is an ongoing search for the holy grail with several candidates available. None of them has reached sufficient sensitivity or specificity. A serum marker of ischemia/reperfusion is HMGB1. Zhang et al. have evaluated this parameter in lower extremity arteriosclerosis and found the postoperative serum levels elevated in patients that would develop restenosis. Although these findings do not relate to ischemia of the intestine, it might, nonetheless, be a future candidate to evaluate successful reperfusion of ischemic intestine. Future exploration of serum parameters has been carried out by Huang et al. who analyzed the postoperative myoglobin levels after liver transplantation. They stated a negative prediction of early allograft dysfunction when myoglobin levels where elevated post operatively.

About 50% of the acute mesenteric ischemias result from embolism of the superior mesenteric artery. In earlier days, open thrombectomy of the embolism was standard of care. Sometimes an often heroic procedure with poor outcome. Even aortomesenteric bypasses have been described, but still not established as standard treatment. Concurrent with general developments in angiographic techniques, the endovascular era captured the visceral arteries and current international recommendations include endovascular revascularization. Lin et al. searched their institutional database on patients who suffered from isolated superior mesenteric artery dissection. They found out that conservative treatment remains a viable option, although endovascular stenting is the better option to secure intestinal perfusion. All of this would depend on the localization of the dissection membrane in order to estimate endovascular success.

